# Sagittal Balance Parameters and Proximal Junctional Kyphosis in Adolescent Idiopathic Scoliosis

**DOI:** 10.3390/jcm13071895

**Published:** 2024-03-25

**Authors:** Galateia Katzouraki, Elias S. Vasiliadis, Angelos Kaspiris, Dimitrios-Stergios Evangelopoulos, Theodoros B. Grivas, Spiros G. Pneumatikos

**Affiliations:** 1 3rd Department of Orthopaedics, School of Medicine, National and Kapodistrian University of Athens, KAT Hospital, 145 61 Athens, Greece; gkatzouraki@med.uoa.gr (G.K.); angkaspiris@upatras.gr (A.K.); dsevangel@med.uoa.gr (D.-S.E.); spirosgp@med.uoa.gr (S.G.P.); 2 Former Head of Department of Orthopedics & Traumatology, “Tzaneio” General Hospital of Piraeus, 185 36 Piraeus, Greece; tgri69@otenet.gr

**Keywords:** sagittal balance parameters, proximal junctional kyphosis (PJK), adolescent idiopathic scoliosis, pediatric deformity, risk factors, spine

## Abstract

**Background:** To review and evaluate multiple preoperative and postoperative sagittal parameters and their association with the risk of developing proximal junctional kyphosis (PJK) in patients with adolescent idiopathic scoliosis (AIS) who undergo correction surgery. **Methods:** A systematic search was performed in December 2022 in PubMed, Embase and the Cochrane Library to retrieve all the studies relevant to our research. After the study selection and data extraction following PRISMA guidelines, RevMan 5.3 was used for statistical analysis. All the analyzed factors were evaluated by using odds ratios and weighted mean differences with 95% confidence intervals. Moreover, the meta-analysis of proportions via MedCalc was used for analyzing quantitative data from the studies. **Results:** A total of 22 studies were included in our meta-analysis. All the available values of sagittal parameters were evaluated. Among all the potential risk factors, higher preoperative thoracic kyphosis (Test for overall effect Z = 11.79, *p* < 0.00001), higher preoperative sagittal vertical axis (SVA) (test for overall effect Z = 11.19, *p* < 0.00001), greater thoracic kyphosis change post-op. compared to pre-op. (test for overall effect Z = 6.02, *p* < 0.00001), increased postoperative lumbar lordosis (test for overall effect Z = 3.65, *p* = 0.0003), higher post-op. SVA (test for overall effect Z = 24.93, *p* < 0.00001) and a larger pelvic incidence/lumbar lordosis (PI/LL) mismatch (test for overall effect Z = 20.50, *p* < 0.00001) were found to be the risk factors for PJK after AIS surgery. Moreover, a decreased rod contour angle (RCA) (test for overall effect Z = 3.79, *p* < 0.0002) and higher proximal junctional angle–rod contour angle (PJA-RCA) (test for overall effect Z = 39.18, *p* < 0.00001) play a significant role in the risk of developing PJK after AIS correction. **Conclusions:** Sagittal balance is of great importance when considering the surgical correction of AIS. Many factors in our meta-analysis were found to increase the incidence for PJK such as higher preoperative thoracic kyphosis and pre-op. SVA. Furthermore, increased thoracic kyphosis change, increased post-operative lumbar lordosis, SVA and PI/LL mismatch are also factors that influence the possibility of post-op. PJK. Lastly, RCA and PJA-RCA are two important factors that need attention during AIS, as over-contouring of the rod could lead to PJK in AIS patients.

## 1. Introduction

Proximal junctional kyphosis (PJK) is a frequent condition identified after spinal fusion in adults and children. PJK is defined as the abnormal kyphotic deformity that occurs at the upper instrumented vertebra (UIV) of the instrumentation. Lee et al. first studied the incidence of PJK and defined it as the kyphotic angle of more than 5° at the proximal end of instrumentation [1]. Later, Glattes et al. increased “the measured Cobb angle ≥ 10° and at least 10° greater than the pre-operative measurement between the UIV and the cephalad endplate of the two levels above (UIV + 2)”, as the second criterion [2]. There is still no consensus as to the definition of PJK, but most of the studies in the literature use the cut-off value of 15° in the proximal junctional Cobb angle [3,4,5,6,7].

Proximal junctional kyphosis is a controversial issue regarding the clinical significance in adolescents, despite the multiple studies performed. The risk factors and various strategies for the prevention of this complication have been described but not fully elucidated. This systematic review aims to identify the risk factors and develop preventive strategies for PJK after surgical correction of adolescent idiopathic scoliosis.

## 2. Materials and Methods

### 2.1. Literature Search

A systematic review was performed in December 2022 following the PRISMA Statement by searching PubMed, Embase and the Cochrane Library. The MeSH terms “proximal junctional kyphosis” and its corresponding synonyms (postoperative kyphosis, kyphosis) were designated keywords, and the term “Adolescent Idiopathic scoliosis” and its corresponding synonyms (scoliosis, scolio) was combined with an “AND” form for the search strategy.

In our meta-analysis, we only used clinical studies. The literature collection, the quality assessment of the eligible studies and the data extraction were performed by two independent authors. Any dispute was thoroughly discussed.

### 2.2. Selection Criteria

All the included studies met the following inclusion criteria: (i) the original study topic involved children and adolescents (<18 years old) with adolescent idiopathic scoliosis (AIS) who underwent spinal surgery for deformity correction; (ii) the essential information of diagnosis, outcomes and treatment protocols were included; and (iii) the studies were prospective or retrospective cohort studies.

The exclusion criteria were as follows: (i) the study was published in a non-English language; (ii) the study included an identical population.

### 2.3. Quality Assessment

Two authors evaluated the quality of the eligible studies by using Covidence for critical appraisal, based on the Cochrane Risk of Bias tool.

### 2.4. Data Extraction

Two independent authors reviewed and processed the data from the selected studies. The study design, number of participants and demographic factors were described in all the studies. Age at the time of operation, sex, surgical approach for deformity correction, pre-operative and post-operative radiological measurements, and follow-up period were included in all eligible studies. Most of the studies included the type of adolescent idiopathic scoliosis based on Lenke classification.

### 2.5. Statistical Analysis

Review Manager Version 5.3 (The Nordic Cochrane Center, The Cochrane Collaboration, Copenhagen, Denmark) was utilized for the statistical analyses. A summary of individual studies and an explanation of their findings in relation to the pertinent outcomes were included in the qualitative synthesis. Results that were published in at least two studies were subjected to meta-analysis. Inverse-variance random effects models (REMs) estimated the pooled mean differences (MDs) for continuous outcome measures. From the included studies, the medians with interquartile ranges, or means with *p*-values, means and standard deviations (SDs) were obtained. There were 95 percent confidence intervals (CI) provided for each effect size. To test for statistical heterogeneity (α = 0.05), the χ^2^ test was employed, and I^2^ statistics were used to quantitatively assess heterogeneity. The threshold for statistical significance was established at *p* ≤ 0.05. Furthermore, for quantitative data analysis (Lenke type and UIV), meta-analysis of proportions was used by utilizing MedCalc (v. 20.0.1 MedCalc Software Ltd., Ostend, Belgium).

## 3. Results

### 3.1. Literature Search Results

**Figure 1:**
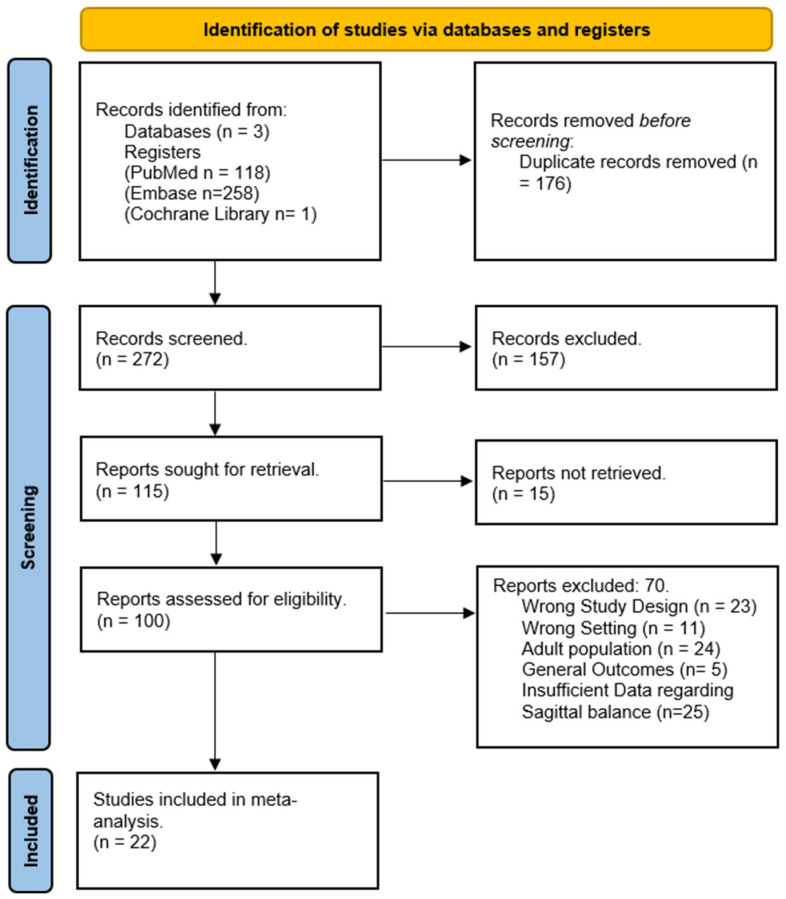
Flow diagram for the systematic review and meta-analysis of the effect of sagittal balance parameters in developing PJK after AIS correction.

The flow chart in Figure 1 illustrates the specifics of the literature search procedure. In total, 189 publications were retrieved from the literature. After eliminating 76 duplicate articles and scanning 113 titles and abstracts, 57 articles were selected for a full-text review. All the study articles were retrieved. Seventeen studies out of the fifty-seven that were chosen for eligibility were disqualified for different reasons. Three studies were excluded because they were conducted in incorrect settings, two of the studies involved an adult population and two of the articles had general outcomes and did not include any risk factors for proximal junctional kyphosis. Eight studies were excluded due to an improper study design and inadequate clinical information. Finally, 40 studies remained for an additional analysis; however, 18 of them lacked enough information about the PJK and non-PJK groups, as well as the sagittal balance parameters. Therefore, 22 studies were analyzed in our meta-analysis. In our systematic review, we included a total of 3922 patients; all were under the age of 18 years and were operated on due to adolescent idiopathic scoliosis [1,4,5,8,9,10,11,12,13,14,15,16,17,18,19,20,21,22,23,24,25,26] (Figure 1).

### 3.2. Demographic Characteristics

In our systematic review, 3922 patients were included. The majority of the patients were females (3222 females versus 700 males), and the mean age of operation was 14.908 years old. The characteristics of the included studies are summarized in Table 1. The incidence of PJK in adolescent idiopathic scoliosis varied from 0% to 46% of the operated cases [1,4,5,6,7].

#### Lenke Classification

**Figure 2:**
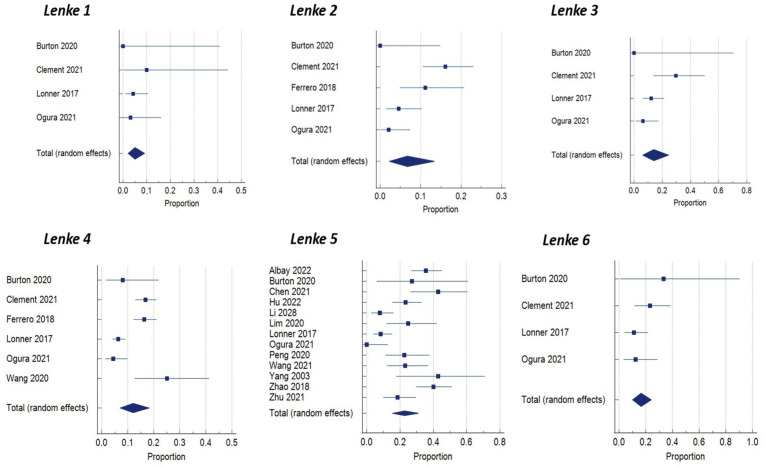

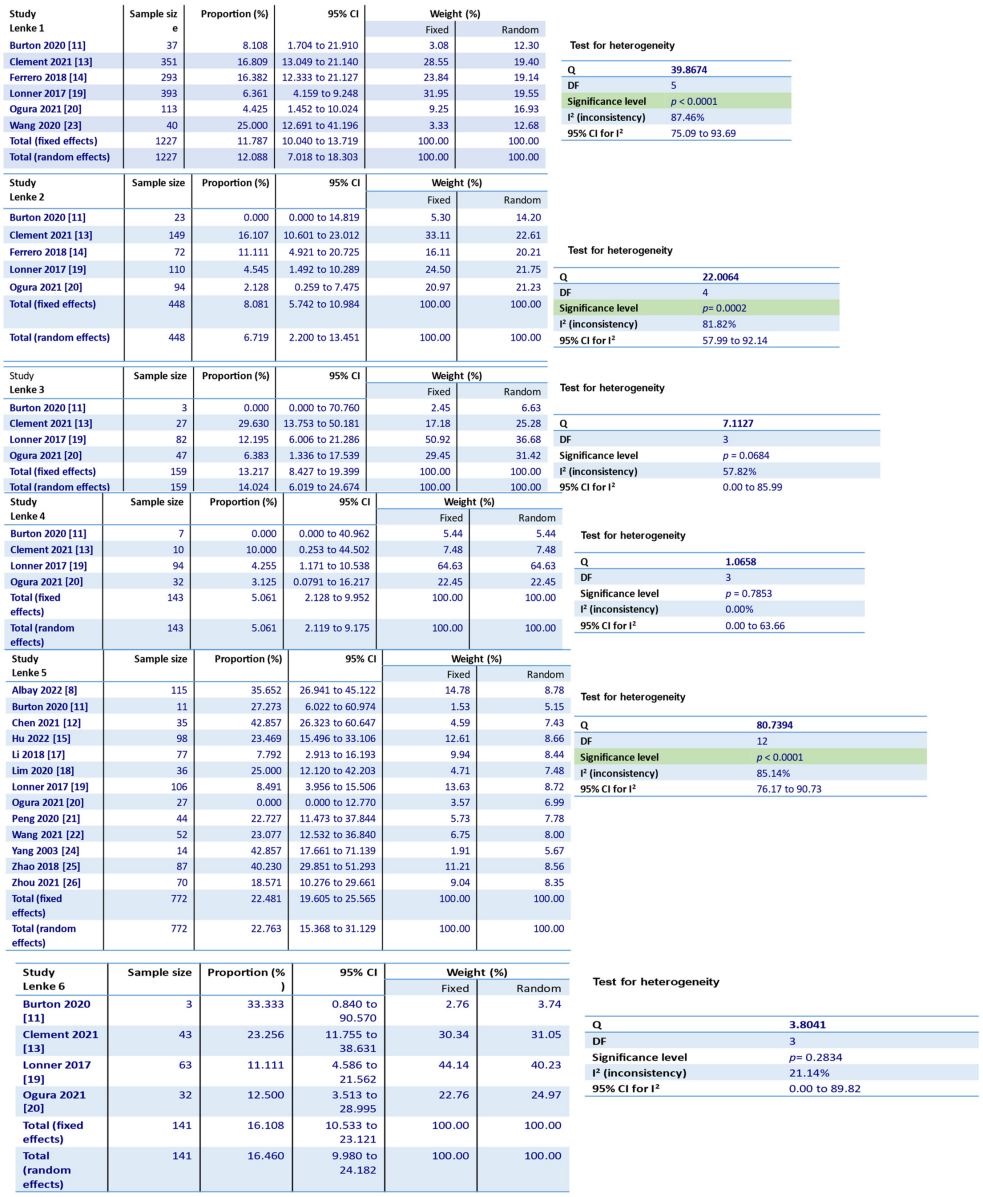
Forest plot of Lenke classifications between the PJK and non-PJK groups, using meta-analysis of proportions.

In the meta-analysis, we analyzed the Lenke classification and the incidence of developing proximal junctional kyphosis after scoliosis correction. According to our results, patients with AIS Lenke type 1, Lenke type 2 and Lenke type 5 have a higher chance of proximal junctional kyphosis following corrective surgery, with a statistically significant difference (Figure 2).

### 3.3. Pre-Operative Sagittal Balance Parameters

#### 3.3.1. Pre-Operative Thoracic Kyphosis

**Figure 3:**
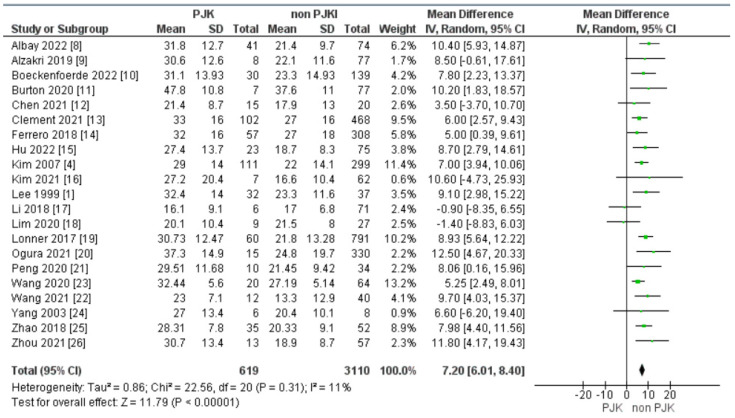
Forest plot of preoperative thoracic kyphosis (TK) angle between the proximal junctional kyphosis (PJK) and non-PJK groups.

Our meta-analysis showed that the pre-operative thoracic kyphosis angle (TKA) was strongly correlated with an increased incidence of post-operative proximal junctional kyphosis. The risk of PJK after the operation for AIS correction was higher when the patients had a larger preoperative TKA (Figure 3).

#### 3.3.2. Pre-Operative Lumbar Lordosis

**Figure 4:**
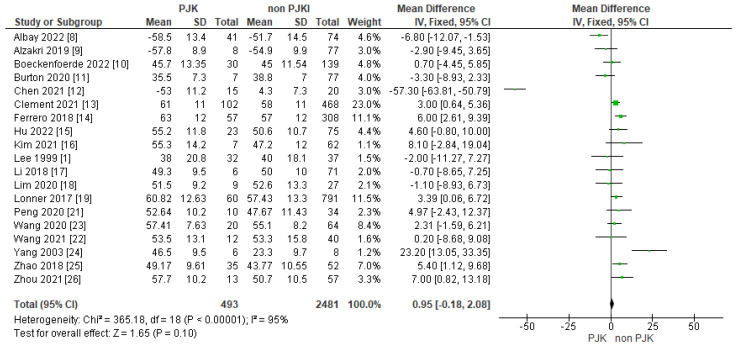
Forest plot for pre-op. LL for the PJK and non-PJK groups.

Pre-operative lumbar lordosis was analyzed in 19 of our included studies. The analysis of the data indicated that pre-operative lumbar lordosis is not a risk factor of PJK after AIS correction (Figure 4).

#### 3.3.3. Pre-Operative SVA and PI/LL

**Figure 5:**
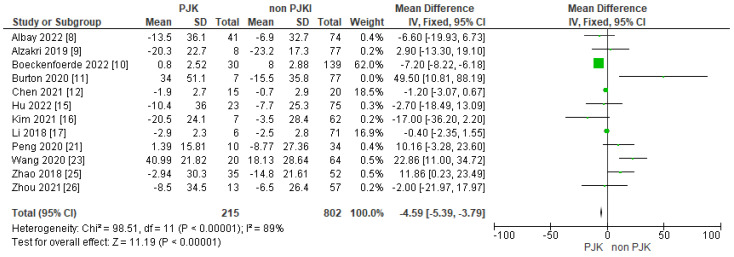
Forest plot for pre-op. SVA for the PJK/non-PJK groups.

**Figure 6:**
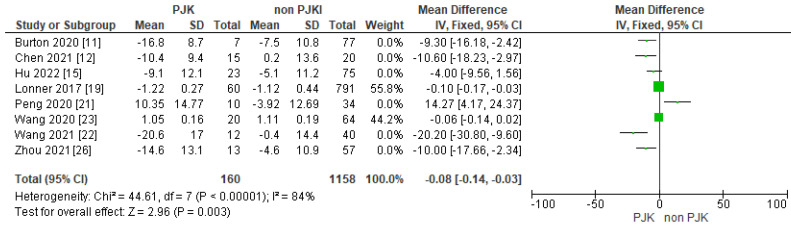
Forest plot for pre-op. PI-LL for the PJK/non-PJK groups.

Regarding the pre-operative SVA, we analyzed 12 studies and our results showed strong correlation between pre-op. SVA and the risk of PJK in AIS (Figure 5). Moreover, eight of our included studies displayed a PI/LL mismatch as a possible factor for post-operative PJK in adolescent idiopathic scoliosis (Figure 6), but with no statistically significant difference.

### 3.4. Post-Operative Sagittal Balance Parameters

#### 3.4.1. Post-Operative Thoracic Kyphosis

**Figure 7:**
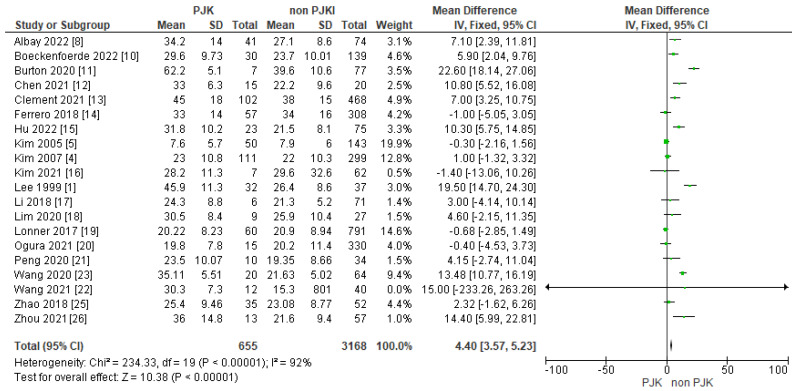
Forest plot for post-operative thoracic kyphosis angle for the PJK and non-PJK groups.

After analyzing 20 of our included studies, we found that post-operative the thoracic kyphosis angle plays an important role in the development of proximal junctional kyphosis after AIS correction (Figure 7).

#### 3.4.2. Thoracic Kyphosis Change

**Figure 8:**
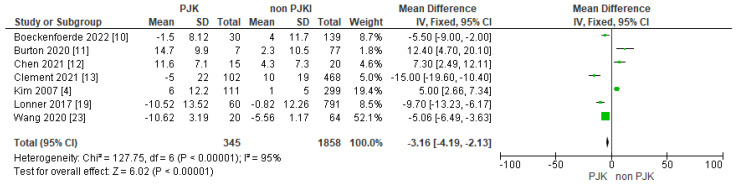
Forest plot for thoracic kyphotic angle change for the PJK and non-PJK groups.

Furthermore, seven of the studies in the meta-analysis indicated that the difference between pre-operative and post-operative thoracic kyphotic angle (TK change) is important for the risk of PJK. The studies showed that if the TK change is high, the risk of PJK after AIS correction is also high (Figure 8).

#### 3.4.3. Post-Operative Lumbar Lordosis (Post-Op. LL)

**Figure 9:**
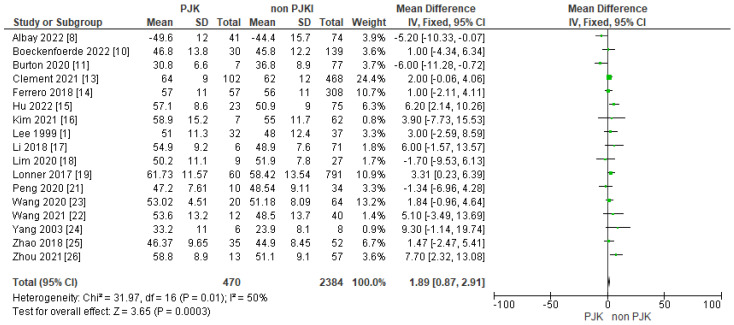
Forest plot for post-operative lumbar lordosis for the PJK and non-PJK groups.

The results of 17 of the included studies indicated that post-operative lumbar lordosis is a significant factor for developing PJK after AIS surgery (Figure 9).

#### 3.4.4. Post-Operative SVA, PI and PI/LL

**Figure 10:**
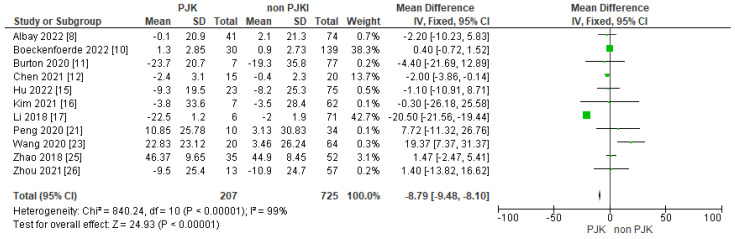
Forest plot for post-operative SVA for the PJK and non-PJK groups.

**Figure 11:**
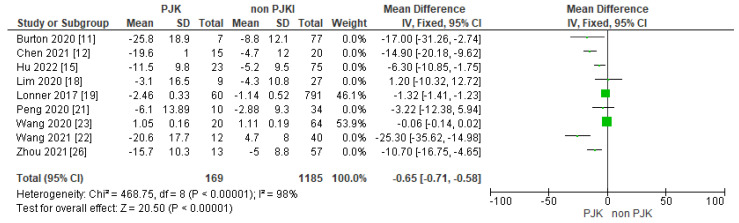
Forest plot for post-operative PI/LL for the PJK and non-PJK groups.

In the analysis, we included post-operative SVA and PI/LL. Both of the parameters seem to be risk factors for developing PJK after AIS correction (Figure 10 and Figure 11).

#### 3.4.5. Post-Operative Proximal Junctional Angle/Rod Contour Angle (PJA/RCA)

**Figure 12:**
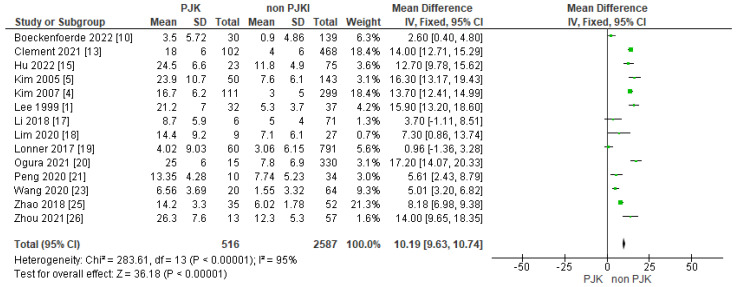
Forest plot for post-operative PJA-RCA angle for the PJK and non-PJK groups.

**Figure 13:**
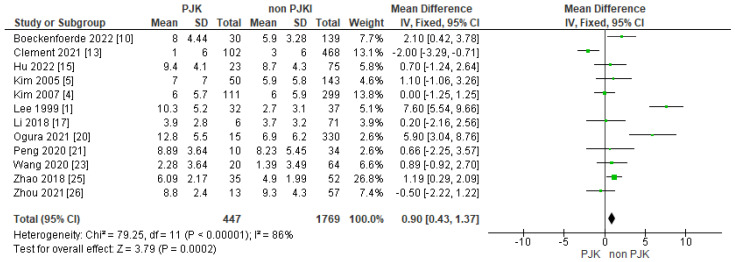
Forest plot for the rod contour angle for the PJK and non-PJK groups.

Of the included studies, 14 displayed their results between the two groups in relation with the proximal junctional angle/rod contour angle and found that is another potential factor for post-operative kyphosis (Figure 12). Moreover, 12 of the studies showed that the rod contour angle is a factor that we need to consider, as the results shows that it is a significant parameter in the development of PJK in AIS (Figure 13).

### 3.5. Peri-Operative Factors

#### Upper Instrumented Vertebra (UIV)

**Figure 14:**
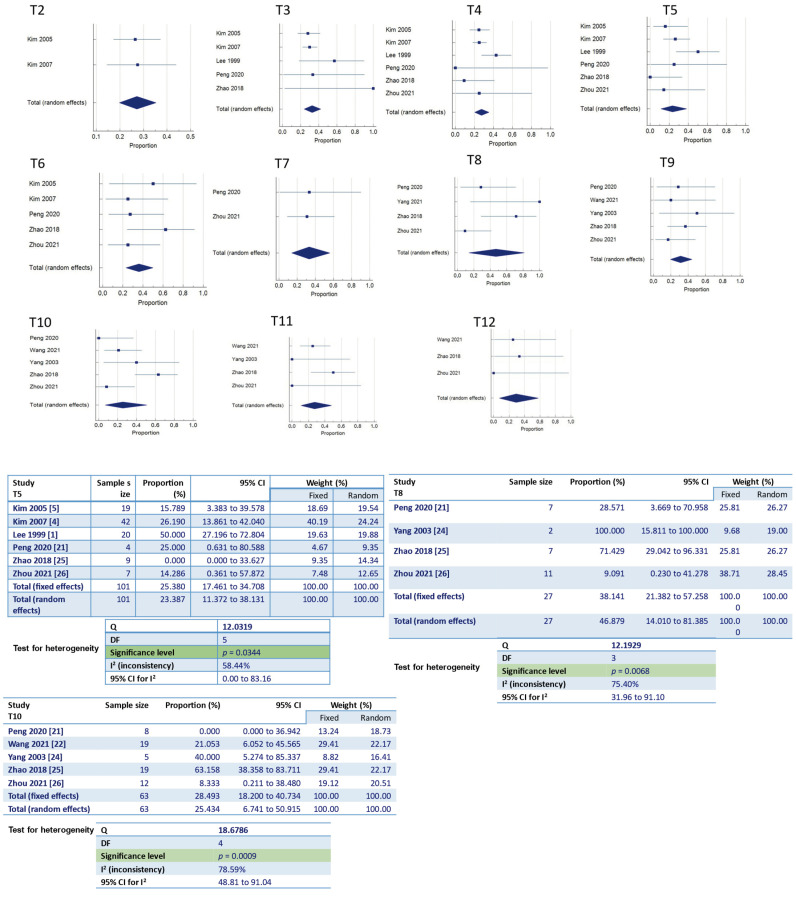
Forest plot of the UIV and proximal junctional kyphosis.

We have analyzed the position of the upper instrumented vertebra (UIV) and the risk of PJK after scoliosis corrective surgery. According to our findings, when the UIV is at levels T5, T8 and T10, there seems to be a higher risk of developing PJK, with a statistically significant difference (Figure 14). All of the studies with the UIV at levels T8 and T10 involved patients with Lenke 5 scoliotic deformities. On the other hand, when the UIV was at T5, most of the studies included patients with AIS Lenke 1 and 2.

## 4. Discussion

Proximal junctional kyphosis is a frequent condition described after the correction of spinal deformity. PJK after adult corrective deformity surgery is well described, and the risk factors and preventive protocols have been extensively reviewed [27]. There are some recent studies evaluating the incidence, risk factors and preventive techniques of PJK after the correction of adolescent idiopathic scoliosis [8,9,10,12,16,23,26,28,29]. The sagittal balance is a parameter that needs to be evaluated thoroughly within the adolescent population with spinal deformities, as it plays an important role in clinical outcomes [30]. In our systematic review, we attempted to outline the sagittal parameters that may lead to PJK in adolescent idiopathic scoliosis patients.

Firstly, there is a need to clarify the definition of PJK in AIS patients. Lee et al. [1] defined PJK as “the measurement of kyphotic angle of higher than 5 degrees than normal at the proximal level of instrumented fusion”. On the other hand, Glattes et al. [2] increased the degrees of the definition to “higher than 10 degrees and the angle measured was of the one composed of the UIV and the upper endplate of UIV + 2” and added that the angle needs to be at least 10 degrees higher than the pre-operative one. Helgeson et al. [3] described PJK as the angle formatted by the UIV and UIV + 1 more than 15 degrees. There is no clarity on the exact definition of PJK, but most of the studies utilized in this systematic review used the definition given by Glattes et al. [2].

### 4.1. Incidence

A fundamental definition that we should always keep in mind is that AIS is a three-dimensional deformity and when we choose to correct it, care must be given, except for coronal balance, to the sagittal balance as well [31]. PJK is a problem related to sagittal balance. The incidence of PJK in AIS throughout the literature varies from 0% to 46% [1,4,5,6,7]. Rhee et al. [6] studied 110 patients and found that 35% of the patients with a posterior approach and fusion had an increase in PJA of more than 10 degrees but no revision surgery was performed. Loner et al. [19], in their prospective multicenter, Level II study, included 851 patients with various types of AIS, and identified an overall incidence of PJK at 7.05% (ranging from 4.39% to 11.64%, depending on the Lenke type of AIS). Clement et al. [13] retrospectively studied 570 patients with AIS and found that 17.89% (102/570 patients) had an abnormal post-operative proximal junctional angle. Kim et al. [4] studied 410 AIS patients treated with three different techniques of posterior instrumentation, and found a PJK incidence of 27% (111/410 patients) with a 2-year follow-up.

### 4.2. Risk Factors

#### 4.2.1. Lenke Classification

Undeniably, the Lenke classification is of great importance in pre-operative planning when a surgical approach is considered for AIS. Lonner et al. [19] showed that in AIS patients, the incidence of post-operative PJK (7.05 percent) was significantly lower than previously reported values. They showed that patients with Lenke 3 and 6 curves had the highest incidence of PJK (11.64%). In our meta-analysis, 17 studies included information regarding the incidence of PJK in correlation with the Lenke classification. Based on our results, we concluded that patients with Lenke type 1, 2 and 5 have a higher incidence of developing PJK post-operatively. Boeckenfoerde et al. [10], in their study, found variation in PJK incidence amongst different Lenke types of AIS, but with no statistically significant difference. Unfortunately, the number of different Lenke type scoliotic deformities included in our study vary considerably, as there was a small number of patients with Lenke types 4 and 6.

#### 4.2.2. Pre-Operative Parameters in Sagittal Balance

##### Pre-Operative Thoracic Kyphosis

In all the included studies, pre-operative thoracic kyphosis was a crucial sagittal parameter that could influence the development of PJK post-operatively. Loner et al. [19], in their prospective multicenter database study, evaluated 851 patients with AIS and studied the risk factors of PJK and categorized the patients based on the Lenke type. Pre-operative T5-T12 kyphosis was significantly correlated with PJK in patients with Lenke 3 and 6 AIS, when the kyphosis is increased by one degree the possibility of PJK is raised by 5%. Clement et al. [13] retrospectively studied 570 AIS patients and found higher global thoracic kyphosis rates pre-operatively in patient with PJK (102/570), with the mean values of 33° pre-op. TK in the PJK group vs. 27° in the non-PJK group. Zhao et al. [25] found higher pre-operative thoracic kyphosis in the PJK group (*p* < 0.001) (mean values: pre-TK 28.31 PJK vs. pre-TK 20.33 non-PJK). Zhou et al. [26] studied 70 patients with Lenke 5 scoliosis and reported that the PJK group had higher pre-op. TK and pre-op. GTK compared to the non-PJK group (30.7 TK in the PJK group vs. 18.9 TK in the non-PJK group/GTK 37.6 PJK vs. 26.2 non-PJK). Ferrero et al. [14] also found that AIS patients with a higher C7 slope, and therefore higher TK angle, had a higher chance of developing proximal junctional kyphosis after the correction of the deformity.

##### Pre-Operative Lumbar Lordosis, SVA and PI/LL

The global sagittal balance pre- and post-operatively has gained attention in the recent years not only in adult spinal deformity, but in adolescents as well. Recent studies have analyzed the sagittal balance parameters such as LL, PI, PT and PI/LL, and their connection to PJK in patients with the correction of AIS. Yang et al. [24] studied 13 patients with AIS and found that all the patients who developed PJK had a pre-op. LL of more than 35 degrees. Erkilinc et al. [32] in their meta-analysis showed that pre-operative lumbar lordosis is an important factor in developing PJK post-operatively (grade of recommendation B). In their study, Ferrero et al. [14] reported that pre-operative PI and LL were higher in the affected (PJK) group (PI: 57° PJK vs. PI:51° non-PJK/LL: 63° PJK vs. 57° non-PJK). Wang et al. [22] investigated the correlation between PI and sagittal pelvic parameters in 52 Lenke 5 AIS patients and PJK. They found that in the patients with kyphosis, there was a significantly lower PI (*p*: 0.016), lower PI/LL mismatch (*p*: 0.022) and lower LL/PI ratio than the non-PJK group (32.9° ± 11.1 vs. 52.9° ± 17.4, − 20.6° ± 17.0 vs. − 0.4° ± 14.4 and 0.64 ± 0.22 and 1.02 ± 0.35, respectively). Chen et al. [12] reported lower a pre-op. PI (mean: 42.6 PJK 50.5 non-PJK *p*: 0.012) and lower PI-LL (mean: −10.4 PJK, 0.2 non-PJK, *p*: 0.014) in the PJK group than in the non-PJK group. Furthermore, Zhao et al. [25] found that the patients who developed PJK in their study group had a significantly higher LL pre-operatively (mean 49.17) compared to the non PJK ones (mean 43.77) (*p*: 0.017). Clement et al. [13] found that global LL was considerably increased in the PJK group (mean: 61°) versus the non-PJK group (mean: 58°, *p*: 0.006). Zhou et al. [26] reported similar results, with a higher pre-operative LL (mean: 57.7°) and lower PI-LL (mean −14.6) in PJK patients than those who did not develop PJK (LL: 50.7 and PI-LL −4.6). In our meta-analysis, pre-operative lumbar lordosis did not show a significant impact on developing post-operative PJK. Moreover, a PI/LL mismatch did not show a statistically significant difference in developing PJK based on our analysis (test for overall effect Z = 2.98, *p* = 0.003). On the other hand, pre-operative SVA seems to be a significant risk factor for developing PJK after AIS correction (SVA: test for overall effect Z = 11.19, *p* < 0.00001).

#### 4.2.3. Post-Operative Sagittal Parameters

##### Post-Operative Thoracic Kyphosis and Change in Kyphotic Angle

Maruo et al. [33] in their study of long constructs in the adult population confirmed that an ideal global alignment is the most important preventative factor for PJK. In this analysis, 20 of the included studies demonstrated the significance of post-operative thoracic kyphosis for the protection against developing PJK after AIS correction. Ferrero et al. [14] showed that a reduced post-operative thoracic kyphotic angle compared to the pre-operative measurements leads to an increase in the thoracic kyphosis at the non-instrumented upper junction, and therefore in developing proximal junctional kyphosis. Clement et al. [13], in their study, explained that if the thoracic kyphosis is insufficient compared to the other sagittal parameters post-operatively, the patients will try to attain symmetry by increasing the kyphosis in the proximal non instrumented spine; thus, these patients will develop PJK. Alzakri et al. [9] in their study presented similar results; their patients had balanced sagittal parameters compared to pre-operative measurements either by having sufficient instrumented thoracic kyphosis or by developing PJK when the instrumented TK was not enough. Lee et al. [1] were the first to describe that the greater the post-operative thoracic kyphosis change is, the higher the chance of developing PJK as a compensatory mechanism. Many studies followed to confirm the results of Lee’s team [4,7,19]. Wang et al. [7] indicated that a post-operative reduction of >5° in thoracic kyphosis considerably magnifies the potential of PJK at follow up.

Clement et al. [13] analyzed that every human has a unique TK, based on the formula “PSTK = 2(PT + LL − PI)”, in their retrospective study. In the case of an insufficient TK, the patient may experience PJK and/or distal cervical kyphosis and regain balance by moving the proximal part of the spine above the fixation. The above formula is validated in AIS patients but has not been used for long follow-ups in other studies, and its accuracy in adult patients is debatable [34]. The decompensation of sagittal balance after selective thoracic and thoraco-lumbar fusions is less common in patients with kyphosis of 23 degrees or more, according to a retrospective analysis of 86 patients with Lenke 1 and 2 curves conducted by Rothenfluh et al. [35]. There is no consensus as to what normative sagittal alignment in the adolescent population is; therefore, further studies are needed to define the postoperative patient specific balance required to prevent complication such as PJK.

##### Post-Operative Lumbar Lordosis, SVA and PI/LL Mismatch

In our meta-analysis, 17 studies included showed that post-operative lumbar lordosis is a significant factor in developing PJK in AIS patients. Sun et al. [36], in patients with TL/L scoliosis (Lenke 5), found that a post-operative increased lumbar lordosis resulted in PJK at follow-up. Moreover, Kim et al. [4] showed that increased post-operative lumbar lordosis was found in patients with PJK who required surgical correction. There are some studies that contradicts our results and observed no strong correlation between increased postoperative lumbar lordosis and PJK in AIS patients [6,37].

Another sagittal parameter with a significant impact on developing PJK is post-operative high SVA. Of the studies included, 11 mentioned post-operative SVA as a parameter that could be a risk factor for PJK [8,10,11,12,15,16,17,26] with statistically significant difference (overall effect Z = 24.93, *p* < 0.00001). Angelliaume et al. [38] found that a higher post-operative SVA (SVA posterior shift) leads to an increased chance of PJK in patients with AIS correction. Sun et al. also had similar results [36].

A post-operative PI/LL mismatch is also a parameter that could influence the possibility of PJK development. Nine studies showed a strong correlation between a higher post-op. PI/LL mismatch and the odds of PJK [11,12,15,18,19,21,22,23,26].

##### Post-Operative PJA-RCA and Rod Contour Angle

Rod contour peri-operatively is a significant parameter in the radiological outcome of the correction, but we should not forget its effect in the biomechanical forces throughout the instrumented spine, in the proximal and distal unistrumented junctional areas. In our meta-analysis, 14 of the included studies showed that an increased proximal junctional angle–rod contour angle is a potential risk factor for PJK after scoliosis correction [1,4,5,10,13,15,17,18,19,20,21,23,25,26]. Both rod contour angle (RCA) and PJA-RCA indicate the rod curve, a straight rod curve has a decreased RCA, and a higher PJA-RCA indicates a straight curve compared with the proximal junctional area. Cammarata et al. [39], in their biomechanical study, found that by decreasing the global sagittal rod contour, the PJA and the proximal flexion forces are also reduced; therefore, they minimize the risk of PJK. The results of our meta-analysis are consistent with Dubousset’s theory of “cone of economy”, whereby post-operatively, the spinal alignment tends to balance itself in the position that needs minimal energy. Wang et al. [23], in their retrospective analysis, found that in the PJK group, the value of PJA-RCA was higher than 5° compared to the other (non-PJK) group. Boeckenfoerde et al. [10] found that a one degree rise in RCA results in a 1.3 times increase in PJK potential; this indicates that overbending of the rod in the proximal region should be avoided.

##### Upper Instrumented Vertebra (UIV)

There is much discussion about choosing the proper upper instrumented level. Multiple studies show contradictory results. Kim et al. [4] found that no significant difference was found in the prevalence of PJK at the UIV where fusions ended in the proximal thoracic spine. Li et al. [17], in their study with Lenke 5 AIS, showed that the distal thoracic UIV is a parameter that could lead to PJK. Zhao et al. [25] reached the same conclusion in their study with Lenke 5C AIS patients. Our meta-analysis showed that in Lenke 5 AIS, when the UIV is in the distal thoracic region (T8, T10), the incidence of PJK is higher with a statistically significant difference. Moreover, our results indicate that UIV at T5 could lead to the development of PJK.

The selection of UIV is important for shoulder balance, risk of proximal curve progression, and as we have showed for development of PJK in AIS patients. Surgical technique, extent of fusion and the type of instrumentation for UIV are of great importance. Cammarata et al. [39] found a reciprocity between posterior ligament dissection and increase in stress, which ultimately results in PJK. The preservation of the spinous process of UIV and UIV + 1 with their posterior ligaments is common practice in AIS correction. Boeckenfoerde et al. [10] showed that there was no difference between the two groups in terms of the quantity or degree of removed spinous processes with the posterior ligaments within the fused levels.

Another debatable issue is the extend of instrumentation and its impact on the development of PJK in AIS patients. Kim et al. [4] reported positive correlation between the two groups and the number of fused vertebrae. According to their findings, longer constructs could lead to a higher incidence of PJK. Lonner et al. [19] concluded that in the PJK group, the extent of instrumentation was longer compared to the non-PJK group.

The type of instrumentation of UIV in AIS correction is a controversial topic. Helgenson et al. [3] showed that the group of pedicle screws had a higher possibility of PJK development compared to the group of hybrid or only hooks. Kim et al. [4] found that the prevalence of PJK was higher when using pedicle screw instrumentation in comparison to the hook-only instrumentation. A higher incidence of PJK was associated with increased junctional stress and overall rigidity of pedicle screws when compared to hooks [4,5]. Moreover, Tharwani et al. [40], in their biomechanical study, indicated that in comparison to pedicle screws, the use of hooks in UIV produced a softer landing at the UIV and a gentler transition to normal motion. Contradictory results were presented in the study of Ferrero et al. [14] and in the study of Pahys et al. [41]. Both studies showed that using different anchors (pedicle screws or hooks) in the proximal part of instrumentation for AIS correction did not affect the incidence of PJK.

##### Minimizing the Risk of PJK in AIS: Clinical Suggestions

1. Careful pre-operative planning is needed when AIS correction is considered. Measuring the pre-operative thoracic kyphosis, lumbar lordosis, SVA and PI/LL mismatch are valuable to plan and achieve the optimal sagittal alignment for each patient postoperatively. Patient-specific thoracic kyphosis (“PSTK = 2(PT + LL − PI)”) tends to be the target postoperative thoracic kyphosis after AIS correction. The extent of instrumentation, the surgical approach, the UIV and the rod contour is suggested to be decided in line with the PSTK.
2. Careful selection of fusion levels, type of instrumentation especially at the upper end of the construct, surgical technique and sparing of the spinous processes, their posterior ligaments and posterior facet capsules of the upper instrumented levels (UIV, UIV + 1) could add to diminish the prevalence of PJK after AIS correction.
3. Rod contouring is crucial to minimize the risk of PJK. The maintenance of an individual’s coronal and sagittal spinal alignment post-operatively is the key for avoidance of PJK.

##### Limitations

In this meta-analysis, we had several limitations. Most of the studies included were retrospective studies; therefore, the credibility of the results might be affected. Most of the studies were taken place in different treatment centers, so there is heterogeneity regarding surgical indications, technology and surgical methods between the patients. There are various risk factors for PJK in AIS according to the literature that we did not include in our study as we focused on the sagittal balance parameters preoperatively and postoperatively only. The surgical approach (posterior, anterior, combined), the different instrumentation used in UIV (hooks, screws, hybrid construct), the length of the fusion and the demographics of the patient (age, sex, BMI, type of scoliosis) were not included in our analysis.

Despite the above limitations, this study expands our understanding regarding the significant role of sagittal balance and its parameters pre-operatively and post-operatively for the avoidance of complications such as PJK. Strategies could be developed for better understanding in individual correction of deformity and how surgeons could achieve the best clinical outcome and a balanced spine in the adolescent population. More studies are needed to define what is an ideal sagittal balance in adolescents.

## 5. Conclusions

This meta-analysis indicated the significance of the sagittal balance in proximal junctional kyphosis, a very frequent complication in AIS correction. Pre-operative and post-operative parameters play a critical role in the development of PJK and should be considered in the treatment strategy of AIS patients. Patients with AIS with an increased pre-operative thoracic kyphosis angle and higher SVA are at risk of PJK after their correction. Moreover, a decreased post-operative thoracic kyphotic angle (compared to pre-op. measurements), higher thoracic kyphosis change between pre- and post-op., higher post-operative lumbar lordosis, increased SVA and PI/LL mismatch lead to a higher chance of PJK in AIS patients. Lastly, RCA and PJA-RCA are significant factors that could lead to PJK after AIS correction surgery. Spinal surgeons should always have a pre-operative plan and address all the above-mentioned parameters based on the patient’s needs to avoid complications.

## Figures and Tables

**Table 1:** Characteristics of the studies included in the meta-analysis.

Study	Country	Patients	Male	Female	Age	Surgical_Intervention	PJK	Non_PJK	FU
**Albay 2022** [8]	Turkey	115	17	98	14.6	Posterior Instrumented Fusion	41	74	24 m
**Alzakri 2019** [9]	France	85	10	75	15.6	Posterior Instrumented Fusion	8	77	Min 24 m
**Boeckenfoerde 2022** [10]	Germany	169	21	148	14.7	Posterior Instrumented Fusion with Screws	30	139	27 m
**Burton 2020** [11]	USA	84	26	58	15	Posterior Instrumented Fusion with Screws	7	77	Min 34 m
**Chen 2021** [12]	China	35	5	30	15.7	Posterior Instrumented Fusion with Screws	15	20	Min 24 m
**Clement 2021** [13]	France	570	88	482	15	Various	102	468	49 m
**Ferrero 2018** [14]	France	365	53	312	15	Posterior Instrumented Fusion with Screws	57	308	Min 24 m
**Hu 2022** [15]	China	98	21	77	15.6	Posterior Instrumented Fusion	23	75	24 m
**Kim 2007** [4]	USA	410	73	337	14.7	Posterior Instrumented Fusion	111	299	24 m
**Kim 2021** [16]	Korea	69	7	62	14.2	Posterior Instrumented Fusion	7	62	Min 60 m
**Lee 1999** [1]	USA	69	8	61	14.5	Posterior Instrumented Fusion	32	37	Min 24 m
**Li 2018** [17]	China	77	9	68	14.75	40 Anterior/37 Posterior	6	71	Min 65.2 m
**Lim 2020** [18]	Singapore	36	0	36	14.2	25 Anterior/11 Posterior	9	27	120 m
**Lonner 2017** [19]	USA	851	183	668	14.4	Posterior Instrumented Fusion	60	791	24 m
**Ogura 2021** [20]	USA	345	68	277	14.5	Posterior Instrumented Fusion	15	330	Min 12 m
**Peng 2020** [21]	China	44	10	34	18	Posterior Instrumented Fusion	10	34	Min 12 m
**Wang 2020** [23]	China	84	25	59	14.63	Posterior Instrumented Fusion	20	64	24 m
**Wang 2021** [22]	China	52	11	41	14	Posterior Instrumented Fusion	12	40	Min 24 m
**Yang 2003** [24]	Taiwan	14	1	13	15.8	Posterior Instrumented Fusion	6	8	Min 24 m
**Zhao 2018** [25]	China	87	21	66	13.51	Posterior Instrumented Fusion	35	52	Average 56 m
**Zhou 2021** [26]	China	70	16	54	15.3	Posterior Instrumented Fusion	13	57	Min 24 m
**Kim 2005** [5]	USA	193	27	166	14.3	Posterior Instrumented Fusion	50	143	Min 60 m

## Data Availability

Data available in a publicly accessible repository. The data presented in this study are openly available in FigShare at doi: 10.6084/m9.figshare.25467643.
